# Characterization of a Novel Male Pheromone Compound in *Leucoptera sinuella* (Lepidoptera: Lyonetiidae) and Its Role in Courtship Behavior

**DOI:** 10.3390/insects16010032

**Published:** 2024-12-31

**Authors:** Laura Sánchez-Aros, Abel F. O. Queiroz, Jorge Guajardo, Wilson Barros-Parada, Glenn P. Svensson, Jan Bergmann

**Affiliations:** 1 Institute of Chemistry, Pontificia Universidad Católica de Valparaíso, Valparaíso 2340000, Chile; 2 Center for Biological and Health Sciences, AGES University Center, Avenida Universitária, 23-Parque das Palmeiras, Paripiranga 48430-000, BA, Brazil; 3 Centro Tecnológico del Álamo (Poplar Technology Center), Universidad de Talca, Talca 3460000, Chile; 4 Agronomy School, Pontificia Universidad Católica de Valparaíso, Quillota 2340000, Chile; wilson.barros@pucv.cl; 5 Department of Biology, Lund University, SE-223 62 Lund, Sweden; glenn.svensson@biol.lu.se

**Keywords:** (*Z*)-3-decenyl hexanoate, *Leucoptera sinuella*, mating behavior, male pheromone

## Abstract

The poplar moth, *Leucoptera sinuella* (Lepidoptera: Lyonetiidae), is widely distributed across Europe, Asia, and parts of Africa. It was first identified in Chile in 2015 and has since become a significant pest in the agricultural sector. Additionally, economic losses are further aggravated by the presence of *L. sinuella* pupae in nearby fruit orchards. This study investigated the presence of a male-produced sex pheromone in *L. sinuella*, focusing on the hairpencil (HP) glands, which are known as dissemination structures for male pheromones in lepidopterans. Male HP glands were solvent-extracted and analyzed by gas chromatography–mass spectrometry. Chemical microderivatization and comparisons of mass spectra and retention indices of natural compounds with synthetic standards led to the identification of two compounds: (*Z*)-3-decenyl hexanoate (the major component) and (*Z*)-3-decen-1-ol (a minor component). Although electrophysiological assays did not show detectable antennal responses to (*Z*)-3-decenyl hexanoate, behavioral bioassays demonstrated its role as a short-range courtship signal in *L. sinuella*. Males with ablated hairpencils exhibited significantly reduced courtship success compared to controls; however, exposure of females to synthetic (*Z*)-3-decenyl hexanoate or HP extract restored male courtship success. To our knowledge, (*Z*)-3-decenyl hexanoate has not been previously reported as a sex pheromone component in any insect species, making this discovery an intriguing addition to the diversity of chemical communication in insects.

## Simple Summary

This study addresses chemical communication in the poplar moth, a pest that damages poplar trees in Europe, Asia, Africa, and South America, where it was first detected in Chile in 2015. Since then, it has become a major pest in poplar nurseries and plantations, reducing tree yields and contaminating nearby fruit crops with its pupae. The research aimed to identify the chemical signals or pheromones that male and female moths use to attract each other, which are critical to their mating process. By understanding these signals, researchers hope to develop tools to control moth populations. This study discovered two unique compounds produced by male moths, one of which is critical for attracting females at close range during courtship. When this compound was removed, male moths were far less successful in courtship, but exposure to a synthetic version restored their success. These findings reveal new insights into the complex communication systems of moths, highlighting the chemical diversity involved in their mating behavior. Understanding these interactions contributes to our broader knowledge of insect ecology and could lead to environmentally friendly approaches to managing pest species, ultimately supporting healthier ecosystems and sustainable agricultural practices.

## 1. Introduction

The moth *Leucoptera sinuella* (Reutii) is found throughout Europe, Asia, and parts of Africa. It was first reported in South America in 2015 near Talagante, Chile [1], and has since become a significant pest in nurseries and poplar plantations. This species damages leaves, resulting in reduced yields and impacting plantation productivity. Additionally, its larvae may disperse to nearby orchards, posing a phytosanitary risk for stone and pip fruit trees. While *L. sinuella* does not feed on the fruits themselves, it is regarded as a quarantine pest in several countries like Mexico and the USA due to its potential threat to forestry. Current control measures include removing nearby poplar trees and evaluating insecticide applications [2].

Recently, 3,7-dimethylpentadecane was identified as the main component of the female-produced sex pheromone, with 3,7-dimethyltetradecane and 7-methylpentadecane as minor components [3]. This discovery marks an initial step toward developing monitoring and control methods as part of integrated pest management strategies. During the pheromone analysis, the presence of hairpencil (HP) glands in males was observed. In lepidopterans, male courtship pheromones typically released from these specialized glands are known to significantly influence female mate choice. During prolonged copulation, males of some species transfer not only sperm but also nutrients and defensive chemicals to females, collectively referred to as spermatophores [4]. Studying these glands and the associated compounds is crucial for understanding the complex interactions that govern mating behavior and reproductive success in these insects.

Preliminary analysis of HP extracts suggested that these glands contain a male-specific compound, which we considered to be a potential courtship pheromone. Since no male-emitted compound involved in sexual communication in *L. sinuella*, nor in any species of the family Lyonetiidae, is known to date, the aim of this study was to address this knowledge gap by identifying the compounds produced by the HP glands of male *L. sinuella* and assessing their role in female attraction or acceptance during mating.

## 2. Materials and Methods

*Insects.* The pupae of *L. sinuella* were obtained from silk cocoons on the bark and leaves of *Populus* spp., collected at the end of spring and summer (November to February) during the years 2021–2023. They were collected in paper bags and strips of corrugated cardboard from the Plant Health Laboratory at the University of Talca (coordinates: −35.40469444835754, −71.63571978502503); in the Pedregal nursery in La Cruz-Quillota (coordinates: −32.84835995936221, −71.19734647659209); and in Linares (coordinates: −36.0822279, −71.7643328) and Curicó (coordinates: −34.9623214, −71.2559311) in the Maule region. Once collected, the pupae were individually placed in 1.5 mL Eppendorf tubes. Subsequently, a portion was kept under simulated diapause conditions in darkness at 4 °C, while another portion was transferred to controlled conditions of temperature and light (25 °C; 16:8 [L:D]; 60–70% relative humidity) to promote adult emergence. After emergence, adults were sexed under a stereomicroscope [5].

*Extraction of HP Glands.* Extracts of the semiochemicals present in the male dissemination structures were prepared from virgin 1- to 4-day-old males. During the calling and courtship period (2:00 to 4:00 p.m.) and depending on the availability of insects, males were anesthetized by freezing at −20 °C for 20 min. Subsequently, the HP glands were removed after gently squeezing the abdomen. Then, the glands were extracted for 20 min in hexane (10 µL of solvent per gland), and the solvent was transferred to another clean and dry vial to be stored at −20 °C until analysis. A total of 15 different extracts containing 2–8 glands were prepared, which were used for chemical analysis and for behavioral experiments [3].

*Chemical Analysis.* The HP gland extracts obtained in the procedure described above were analyzed using gas chromatography coupled with mass spectrometry (GC-MS). Analysis was performed using a GCMS-QP 2010 Ultra combination (Shimadzu, Kyoto, Japan) equipped with an SBL-5 capillary column (30 m × 0.25 mm id, 0.25 μm film; Restek, Bellefonte, PA, USA) or a Stabilwax capillary column (30 m × 0.32 mm id, 0.25 μm film; Restek, Bellefonte, PA, USA). The analyses were carried out with a temperature program starting at 50 °C and increasing to 270 °C at a rate of 8 °C/min for the SBL-5 column or from 50 °C to 220 °C at a rate of 8 °C/min for the Stabilwax column. The gas chromatograph was operated in splitless mode (sampling time of 0.5 min) with an injector temperature of 200 °C. Helium was used as the carrier gas at a flow rate of 40 cm/s. Electron impact mass spectra were acquired at 70 eV.

Microchemical derivatization reactions, including catalytic hydrogenation [6] and the formation of dimethyl disulfide (DMDS) adducts [7], were performed on HP extracts to confirm the presence and configuration of double bonds. Detailed procedures are available in the Supplementary Material.

*Chemicals*. (*Z*)-3-Decenyl hexanoate and (*Z*)-3-decen-1-ol were obtained as described below and in the Supplementary Material.

**Figure 1:**
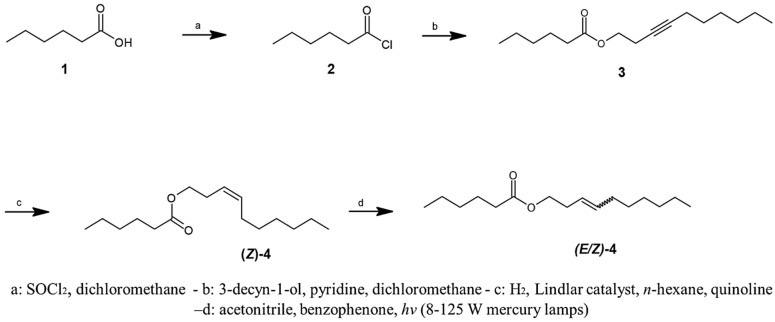
Synthesis of (*Z*)-3-decenyl hexanoate and a stereoisomeric mixture.

*Synthesis of 3-Decenyl Hexanoate.* The synthetic procedures are detailed in the Supplementary Material. In summary, hexanoic acid (1) was first converted to its acid chloride (2), which was then reacted with 3-decyn-1-ol to yield ester 3 (Figure 1). Stereoselective catalytic hydrogenation of the triple bond using Lindlar catalyst subsequently afforded the target compound, (*Z*)-4, with an overall yield of 65%. To confirm the double bond configuration in the natural compound, compound **4** was subjected to a photoisomerization reaction, resulting in an equal mixture of (*E*)- and (*Z*)-3-decenyl hexanoate.

*Electrophysiology.* Electroantennograms (EAGs) were recorded from female *L. sinuella* to check if (*Z*)-3-decenyl hexanoate could trigger a response. An antennal preparation consisted of the head with both antennae and was mounted to a PRG-2 EAG probe (10 × gain) (Syntech, Kirchzarten, Germany) using conductive gel (Blågel, Cefar, Malmö, Sweden). Humidified and charcoal-filtered air was delivered through a glass tube outlet positioned at a 5 mm distance from the antennae. Odor stimuli were prepared by loading a piece of filter paper placed inside Pasteur pipettes with 10 µL aliquots of (*Z*)-3-decenyl hexanoate dissolved in heptane, producing six different doses of the test compound (0.1–10,000 ng). A Syntech Stimulus Controller CS-55 (Syntech) was used to generate a 0.5 s puff passing through the stimulus pipette, introducing the odor to the airflow to reach the antennae. A dose–response sequence started with the lowest dose and ended with the highest dose, with approximately 20 s between stimulations, and before and after such a sequence, the antennae were stimulated with a control (heptane only applied on filter paper). In total, five antennal preparations were used in the analysis. Response amplitudes (mV) were analyzed using GC-EAD Pro Version 4.1 (Syntech, Kirchzarten, Germany), and mean antennal responses for the different stimulus doses were then compared using ANOVA (SPSS Ver. 27, SPSS Inc., Chicago, IL, USA).

*Behavioral Patterns Related to Courtship and Mating.* To document the courtship and mating behaviors of *L. sinuella*, pairs of virgin males and females were observed in separate trials using transparent Petri dishes (5 cm diameter) equipped with 1 mm ventilation holes on the lid and sides. A total of 30 pairs were recorded individually. The observed behaviors included male wing fanning and touching the female with its antennae, female abdominal curving, approaches or retreats by the female, as well as successful copulations. Field observations showed that *L. sinuella* is active during the daytime. Consequently, behavioral observations were conducted during hours of major activity, specifically between 2:00 p.m. and 5:00 p.m., using a Dino-Lite microscope camera along with a Logitech BRIO 4K ultra-HD.

*Evaluation of the Function of Extracted and Synthesized Compounds in Mate Selection.* Behavioral assays were conducted in a BIOREF climatic chamber from 2:00 p.m. to 4:00 p.m. at a temperature of 21–25 °C, according to the methodology taken and adapted from Hillier et al. (2004) [8]. To prepare for the assays, 2–3-day-old males and females were anesthetized by freezing at −4 °C for 10 min starting at 12:20 p.m. and subsequently manipulated under a stereoscope (HP ablation, antennectomy, or sham operation; see below). They were then acclimated for 1 h in separate Petri dishes before pairing (n = 10 pairs) in individual Petri dishes (5 cm diameter) with 1 mm ventilation holes on the lid and sides. Each pair was observed for 30 min with a Logitech BRIO 4K ultra-HD camera (Logitec. Switzerland). Only insects with full mobility and without signs of impairments other than those as a result of the surgery were included in the assays. To examine whether volatiles from male HP glands, detected by female antennae, influence courtship, acceptance, and/or mating, four treatments were designed:

Treatment 1: Females with Antennal Ablation Paired with Normal Males.

Antennal ablation was performed on anesthetized females by removing each antenna at the base with 8 cm curved Vanna scissors (8 mm tip). Males were anesthetized but only received a sham operation.

Treatment 2: Males with HP Ablation Paired with Normal Females.

For male HP ablation, anesthetized males underwent a procedure in which gentle pressure on the last abdominal segment exposed the HP gland, which was then excised using Vanna scissors. Females were anesthetized and handled similarly but did not undergo ablation.

Treatment 3: Males with HP Ablation Paired with Normal Females in the Presence of HP Extract.

Following HP ablation, males were paired with sham-operated females in Petri dishes containing a 1 cm^2^ piece of filter paper loaded with 10 µL (equivalent to 1 HP gland) of natural HP extract in hexane. After allowing the solvent to evaporate, the filter paper was placed in the Petri dish containing the male–female pair.

Treatment 4: Males with HP Ablation Paired with Normal Females in the Presence of Synthetic (*Z*)-3-decenyl hexanoate.

Males underwent HP ablation, while females were anesthetized and handled without surgery. A 1 µg/mL solution of synthetic (*Z*)-3-decenyl hexanoate in hexane was prepared, and 10 µL was applied to a 1 cm^2^ piece of filter paper. After allowing the solvent to evaporate, the filter paper was placed in the Petri dish containing the male–female pair.

Responses were analyzed based on the frequency of (1) courtship-related behaviors exhibited by males, (2) female acceptance or rejection, and (3) copulation. Control observations documented courtship and mating behaviors of sham-operated *L. sinuella* pairs under the same experimental conditions.

*Statistical Analyses.* The proportions of moths from different treatment groups showing each of the behaviors of courtship, acceptance, and mating were compared using chi-square statistics in Excel Version 16.89.1 for Mac users (© 2010 Microsoft Corporation, Redmond, WA, USA).

## 3. Results

### 3.1. Behavior Patterns Related to Courtship and Mating

#### 3.1.1. Calling Behavior of Females

**Figure 2:**
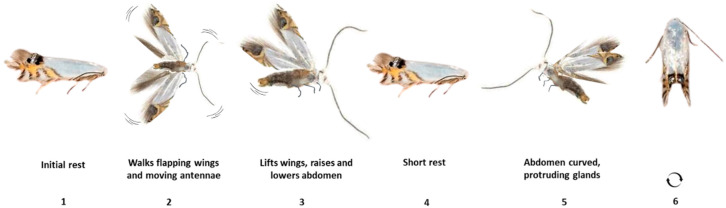
Calling behavior female *L. sinuella*.

Thirty pairs of *L. sinuella* were observed and recorded individually on video to identify behaviors related to calling, courtship, and mating. The analysis revealed a sequence of six distinct, repetitive movements executed by the female (Figure 2): (1) Following vigorous walking throughout the arena, the female paused and remained stationary for 5 to 7 min; (2) The female started fanning the wings, making antennal contact with the arena; (3) The female stopped and elevated the abdomen in an up-and-down motion; (4) Brief rest; (5) The female raised the wings to expose the abdomen, thus protruding the pheromone gland; (6) If the male failed to approach effectively, the female repeated the sequence.

#### 3.1.2. Mating Behavior

**Figure 3:**
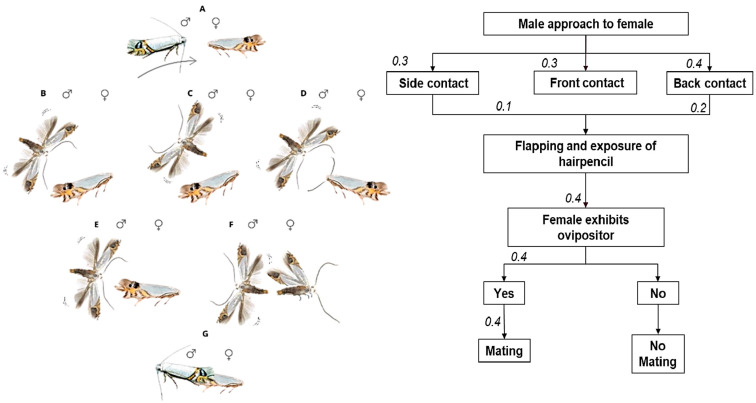
Mating behavior sequence of *L. sinuella*. **Left:** (**A**) male walks towards the female; (**B**) male making side contact; (**C**) male flapping wings towards the female; (**D**) male making frontal contact; (**E**) male making back contact; (**F**) male turns his body and exposes his aedeagus, and female exposes her abdomen; (**G**) copulation. **Right:** Mating ethogram of *L. sinuella* (n = 10 pairs). Values indicate the probability of transition from one passage to the next.

The male initiated mating by approaching the female (Figure 3A) and touching the wings with the antennae from both lateral and frontal positions (Figure 3B,D). During this approach, the male made circular movements, exposing the HP structures and fanning wings vigorously (Figure 3C). Females usually responded by opening the wings and exposing the ovipositor, signaling readiness for copulation (Figure 3F). When direct back contact occurred, the male touched the female’s back with the antennae (Figure 3E), and, in some cases, the female opened the wings and exposed the ovipositor gland directly, thus omitting step C.

If the female opened the wings, the male turned around and inserted the aedeagus into the ovipositor (Figure 3G). In case the female remained unresponsive, the male repeated the courtship behaviors B-F until the female signaled acceptance. Eight of ten males (80%) reached successful copulation. The average copulation time in laboratory conditions was approximately 90 min, during which the female typically remained stationary or moved slowly, accelerating moments before ending copulation to separate from the male.

### 3.2. Chemical Analyses of Gland Extracts

**Figure 4:**
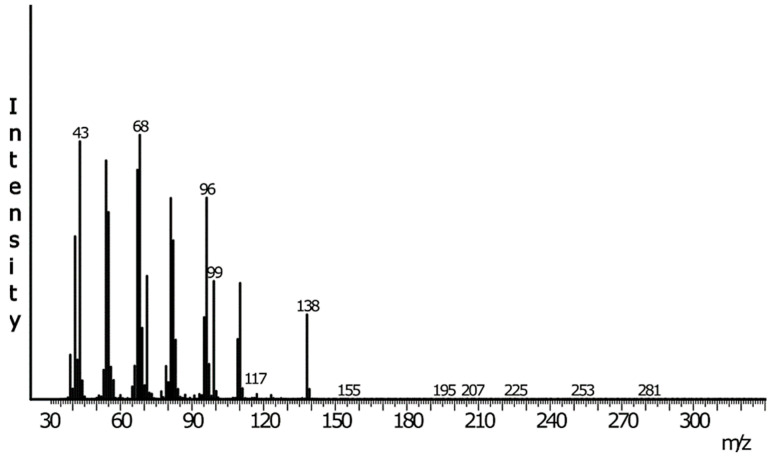
Mass spectrum (70 eV) of compound A in HP gland extracts of male *L. sinuella*.

**Figure 5:**
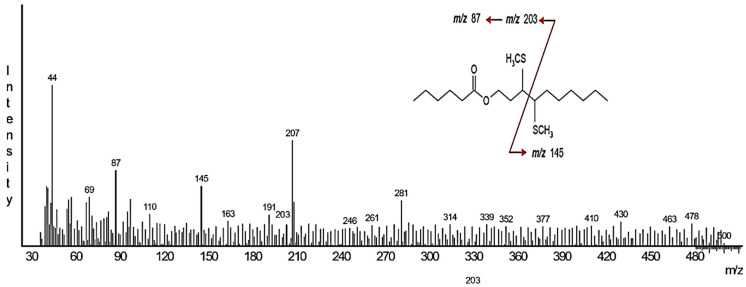
Mass spectrum (70 eV) of the DMDS adduct of compound A. The insert shows diagnostic fragmentation pathways of 3-decenyl hexanoate.

**Figure 6:**
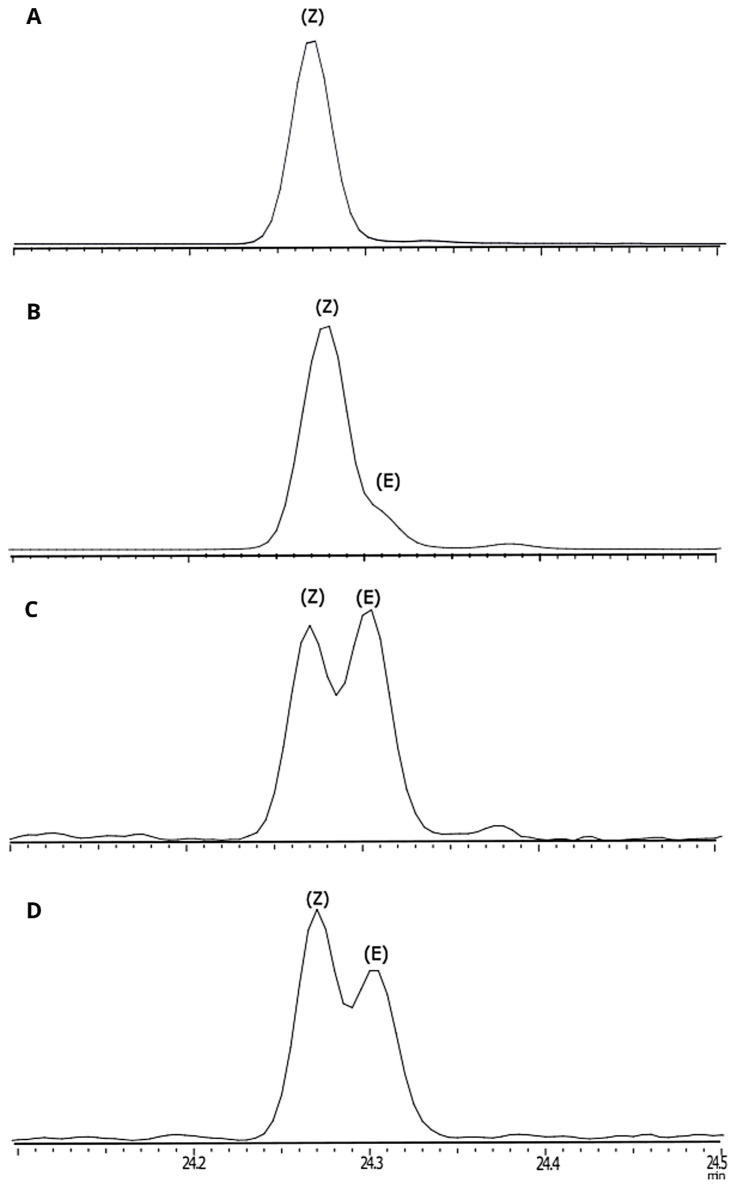
Chromatograms of (**A**) HP extract; (**B**) synthetic (Z)-3-decenyl hexanoate; (**C**) mixture of (*Z*)- and (*E*)-3-decenyl hexanoate after photoisomerization; (**D**) co-injection of HP extract with the mixture of isomers.

GC-MS analyses of HP gland extracts from 1- to 4-day-old virgin males consistently revealed the presence of one major compound (A) and, in some extracts, an additional minor compound (B) (Figure S1). Neither compound was detected in extracts of the rest of the body of males nor in extracts from females. The retention indices of these compounds were 1761 (SLB-5) and 2035 (Stabilwax) for compound A and 1256 (SLB-5) and 1783 (Stabilwax) for compound B. The diagnostic fragments at *m*/*z* 99 and *m*/*z* 117 in the mass spectrum of the main compound A (Figure 4) suggested it to be a hexanoate ester. In this case, the fragment at *m*/*z* 138 would indicate a 10-carbon alcohol moiety with one unsaturation. The resulting putative structure of a decenyl hexanoate is in good accordance with the retention indexes of compound A. Catalytic hydrogenation of the extract and subsequent analysis by GC-MS resulted in the disappearance of compound A and the appearance of a new peak at a slightly higher retention time (RI 1765) (Figure S2). The mass spectrum of this new compound was a perfect match to the database spectrum of decyl hexanoate, further confirming the structure of a decenyl hexanoate for the natural compound. The diagnostic fragments in the mass spectrum of the DMDS adduct of compound A at *m*/*z* 145 and *m*/*z* 203 (the latter loses hexanoic acid to form a strong fragment at *m*/*z* 87) suggested that the double bond was located at position 3 of the decenyl moiety of the original unsaturated ester (Figure 5). Finally, the mass spectrum and the retention times of the natural compound matched those of synthetic (*Z*)-3-decenyl hexanoate but not of the (*E*) isomer, which was confirmed by co-injection of the HP extract and an isomeric mixture of 3-decenyl hexanoate (Figure 6).

Based on the mass spectrum (Figure S3) and retention indexes, and considering the structure of the main ester compound in the HP glands of males, the structure of the minor compound B was likely to be (*Z*)-3-decen-1-ol. This identification was confirmed by matching retention indexes of the natural compound with those of an authentic sample of (*Z*)-3-decen-1-ol on both the polar and non-polar stationary phases (Figure S4).

### 3.3. Electrophysiology

**Figure 7:**
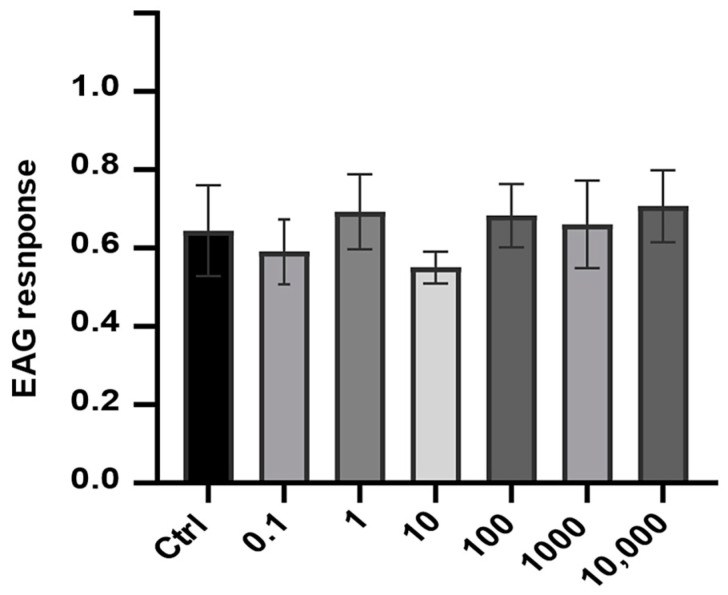
Mean (±s.e.m.) amplitudes of EAG response of female *L. sinuella* antennae to six different doses 0.1–10,000 ng of the test compound (Z)-3-decenyl hexanoate (n = 5).

The EAG recordings showed no significant increase in the response amplitude of antennae of *L. sinuella* females to any of the test doses of (*Z*)-3-decenyl hexanoate when compared with the corresponding response amplitude of the control (F = 0.392, *p* = 0.878; Figure 7). Thus, the observed responses were most likely due to stimulation of mechanosensory receptors on the antennae.

### 3.4. Evaluation of the Function of Extracted and Synthesized Compounds in Mate Selection

#### 3.4.1. Courtship

**Figure 8:**
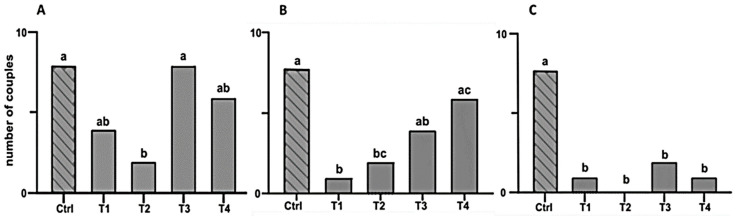
Evaluation of (Z)-3-decenyl hexanoate on mating behavior. (**A**) Male courtship, (**B**) Female acceptance, (**C**) Effective copulation (n = 10). Treatments: Ctrl: Intact, sham-operated pair; T1: female without antenna and normal male; T2: male without HP and normal female; T3: male without HP and normal female, plus HP extract; and T4: male without HP and normal female, plus synthetic (*Z*)-3-decenyl hexanoate. Columns with different letters are significantly different according to the chi-square test (*p* < 0.05).

Courtship behaviors were observed in 8 of 10 couples of intact males and females. Courtship frequency was significantly reduced in pairs of intact females and hairpencil-ablated males (T2, 2 courtships) (χ^2^ = 7.20; d.f. = 1; *p* < 0.01). However, when the hairpencil extract (T3, 8 courtships) or synthetic (*Z*)-3-decenyl hexanoate (T4, 6 courtships) was present, courtship frequency was similar to the control (χ^2^ < 0.95; d.f. = 1; *p* > 0.05). Courtship frequency in pairs with antenna-ablated females and intact males (T1, 4 courtships) showed no statistically significant difference as compared to the control (χ^2^ = 3.33; d.f. = 1; *p* > 0.05) (Figure 8A).

#### 3.4.2. Acceptance

Female acceptance behavior was significantly lower in pairs where either the female (T1, 1 acceptance) or the male (T2, 1 acceptance) was ablated, compared to intact pairs (control, 8 acceptances) (χ^2^ = 7.20–9.90; d.f. = 1; *p* < 0.01). When hairpencil extract (T3, 4 acceptances) or synthetic (*Z*)-3-decenyl hexanoate (T4, 6 acceptances) was introduced, acceptance frequency was restored to control levels (χ^2^ = 0.95–3.33; d.f. = 1; *p* > 0.05) (Figure 8B).

#### 3.4.3. Mating

Effective matings were significantly reduced in all experimental treatments compared to intact pairs (control, 8 matings). Severed female–intact male pairs (T1, 1 mating), severed male–intact female pairs (T2, 0 matings), and severed males paired with intact females plus either HP extract (T3, 2 matings) or synthetic (*Z*)-3-decenyl hexanoate (T4, 1 mating) exhibited significant reductions (χ^2^ = 7.20–13.33; d.f. = 1; *p* < 0.01) (Figure 8C). Additionally, the few matings observed in treatments T1, T3, and T4 were brief (1–3 min) or intermittent.

## 4. Discussion

We have identified (*Z*)-3-decenyl hexanoate as a male-produced pheromone component in *L. sinuella* based on chemical and behavioral analyses. This compound, not previously documented as a pheromone in any insect species, adds to the chemical diversity reported for male-produced sex pheromones in moths. Additionally, we characterized the mating behaviors of *L. sinuella*, distinguishing between calling, courtship, female acceptance, and copulation phases. Finally, we obtained evidence that male HP volatiles, specifically (*Z*)-3-decenyl hexanoate, play an important role in the mating behavior of the species.

Observed mating behaviors align with reports for other Lyonetiidae species, such as *L. coffeella*, where, upon female calling, males approach the female, exposing their HPs with vigorous wing movements from various positions [9]. Female wing opening and ovipositor exposure signal acceptance and readiness for copulation. These behaviors are also comparable to those documented for moths from other families, such as *Chloridea* (formerly *Heliothis*) *virescens* (Noctuidae) and *Grapholita molesta* (Tortricidae), where males display HPs and engage in wing movements to stimulate females [6,8].

The detection of male odor by females apparently plays a crucial role in mating success in *L. sinuella*. This became evident by the behavior of pairs of antennectomized females and normal males (T1), where female acceptance and successful copulation were significantly reduced as compared to pairs of normal females and males. Decreased acceptance of courting males by antennectomized females and lower copulation success have also been observed, for example, in *C. virescens* [8] and *Conogethes punctifiralis* [10]. We also observed reduced acceptance and copulation in *L. sinuella* when HP-ablated males were placed with normal females (T2), showing a statistical difference from the control. These results confirm that HP presence and the delivery of volatile compounds through HP-disseminating structures significantly influence mating behaviors, consistent with reports on male lepidopteran behaviors across families [9,10,11,12,13].

We tested synthetic (*Z*)-3-decenyl hexanoate and natural HP extracts in pairs with surgically removed HP structures. Results showed that male courtship was significantly reduced without HPs, underscoring the role of volatile compounds delivered through HP structures. The decrease in male courtship behavior could be a result of females not initiating calling in the absence of HP volatiles and not necessarily be attributable to the fact that males were severed because male courtship behaviors were restored when pairs of HP-ablated males and normal females were exposed to HP extracts (T3) or synthetic (*Z*)-3-decenyl hexanoate (T4). Similarly, acceptance of males by females was significantly lower in treatments with HP-ablated males but could be restored to control levels when pairs were exposed to synthetic (*Z*)-3-decenyl hexanoate or to HP extract. These findings demonstrate the compound’s significance in female mate selection and its function as a cue that enhances male courtship behaviors. This aligns with previous research conducted on *C. virescens*, *Chloridea subflexa* [8,12], and *Ostrinia nubilalis* [14], which showed that males lacking hairpencils (HPs) experienced reduced mating success with females. However, their mating success could be restored when exposed to HP extracts, indicating the crucial role of volatiles emitted from HPs in facilitating male courtship behavior in these species.

Successful copulation was significantly reduced in all treatments where females or males had received surgical treatment. Unlike male courtship and female acceptance, copulation success was not restored in the presence of HP extract nor synthetic (*Z*)-3-decenyl hexanoate. This is probably due to the manipulation of males, as we observed difficulties for HP-ablated males upon attempting to insert the aedeagus into the ovipositor.

In other Lepidopteran species closely related to each other, male HP compounds play an important role in reproductive isolation. This was demonstrated for *C. virescens*, *C. subflexa*, and *Helicoverpa zea* [8,12]. Whether there is a similar role in *L. sinuella* and its close relatives remains unclear.

Male moth pheromones are chemically diverse and have different biosynthetic origins. An important group is modified host plant volatiles, such as pyrrolizidine alkaloid derivatives (danaidal and related compounds, from erebid moths) or floral and fruit aroma compounds (e.g., methyl 2-epijasmonate from *G. molesta*, 4-hydroxybenzaldehyde from the pyralid *Eldana saccharina*, or 2-phenylethanol from the noctuid *Mamestra brassica*) ([4], and references cited therein). Another group is fatty acid-derived long-chain saturated and unsaturated compounds with an oxygenated functional group (alcohol, ester, or carboxylic acid). The HP-produced (*Z*)-3-decenyl hexanoate in *L. sinuella* falls into this latter group and adds to the diversity of structures of male lepidopteran pheromones, as ester compounds known to date are either acetates or ethyl esters. Furthermore, this is the first example of a male pheromone from a member of the family Lyonetiidae. Examples of other fatty acid-derived male pheromone compounds are hexadecyl acetate and (*Z*)-9-, (*Z*)-11-, and (*Z*)-14-hexadecenyl acetate from *O. nubilalis* as courtship pheromones with a role in mate choice of females [14]. Hexadecyl acetate, together with other structurally related saturated alcohols, acetates, and carboxylic acids with chains of 14, 16, and 18 carbon atoms, was also reported from *C. virescens* [15], and these compounds play a role in mate acceptance and species isolation [8].

A possible biosynthetic route to (*Z*)-3-decenyl hexanoate could start with palmitoleic acid ((*Z*)-9-hexadecenoic acid). Three steps of chain-shortening via a β-oxidation pathway, in each of which an acetate unit is removed, would produce (*Z*)-3-decenoic acid, with (*Z*)-7-tetradecenoic and (*Z*)-5-dodecenoic acids as intermediates [16]. Reduction of (*Z*)-3-decenoic acid would then produce (*Z*)-3-decen-1-ol (found as a minor compound in HP glands), which can be esterified with hexanoic acid to produce the main compound.

No significant electrophysiological response was observed when antennae of female *L. sinuella* were stimulated with various doses of (*Z*)-3-decenyl hexanoate, which could be a result of too few receptors present on the antennae tuned to the male-produced compound to elicit a detectable response. The minute size of *L. sinuella,* making reliable antennal preparations difficult to produce, may also explain the lack of response. The fact that few identification studies on male-produced sex pheromones in moths include results from electrophysiological analyses of candidate compounds suggests that such data are difficult to generate. A few exceptions are the recent studies on *Galleria mellonella* by Svensson et al. (2014) [17] and *Chloridea virescens* by Liu et al. (2023) [18], where strong antennal responses were elicited in females upon stimulation with behaviorally relevant male-derived compounds.

## Data Availability

The original data presented in this study are openly available in FigShare at 10.6084/m9.figshare.27930297.

## Supplementary Materials

The following additional information can be downloaded at: https://doi.org/10.6084/m9.figshare.27930297.v2. A detailed description of the synthesis of (*Z*)-3-decenyl hexanoate and of microderivatization procedures; Figure S1: Gas chromatogram of the hairpencil extract of male *L. sinuella*; Figure S2: Gas chromatogram of the extract obtained from pheromonal glands of male *L. sinuella* and its hydrogenation; Figure S3: Mass spectrum of compound B ((*Z*)-3-decen-1-ol); Figure S4: Chromatograms of synthetic (*Z*)-3-decen-1-ol showing retention indexes on both polar and apolar columns; Example videos showing calling behavior of females and mating behavior of male–female pairs.
